# Beyond the Lesion: Back to High Fidelity DNA Synthesis

**DOI:** 10.3389/fmolb.2021.811540

**Published:** 2022-01-05

**Authors:** Joseph D. Kaszubowski, Michael A. Trakselis

**Affiliations:** Department of Chemistry and Biochemistry, Baylor University, Waco, TX, United States

**Keywords:** DNA replication, polymerase, switching, translesion synthesis, PCNA, DNA damage, DNA lesion, substitution

## Abstract

High fidelity (HiFi) DNA polymerases (Pols) perform the bulk of DNA synthesis required to duplicate genomes in all forms of life. Their structural features, enzymatic mechanisms, and inherent properties are well-described over several decades of research. HiFi Pols are so accurate that they become stalled at sites of DNA damage or lesions that are not one of the four canonical DNA bases. Once stalled, the replisome becomes compromised and vulnerable to further DNA damage. One mechanism to relieve stalling is to recruit a translesion synthesis (TLS) Pol to rapidly synthesize over and past the damage. These TLS Pols have good specificities for the lesion but are less accurate when synthesizing opposite undamaged DNA, and so, mechanisms are needed to limit TLS Pol synthesis and recruit back a HiFi Pol to reestablish the replisome. The overall TLS process can be complicated with several cellular Pols, multifaceted protein contacts, and variable nucleotide incorporation kinetics all contributing to several discrete substitution (or template hand-off) steps. In this review, we highlight the mechanistic differences between distributive equilibrium *exchange* events and concerted contact-dependent *switching* by DNA Pols for insertion, extension, and resumption of high-fidelity synthesis beyond the lesion.

## Introduction

**FIGURE 1:**
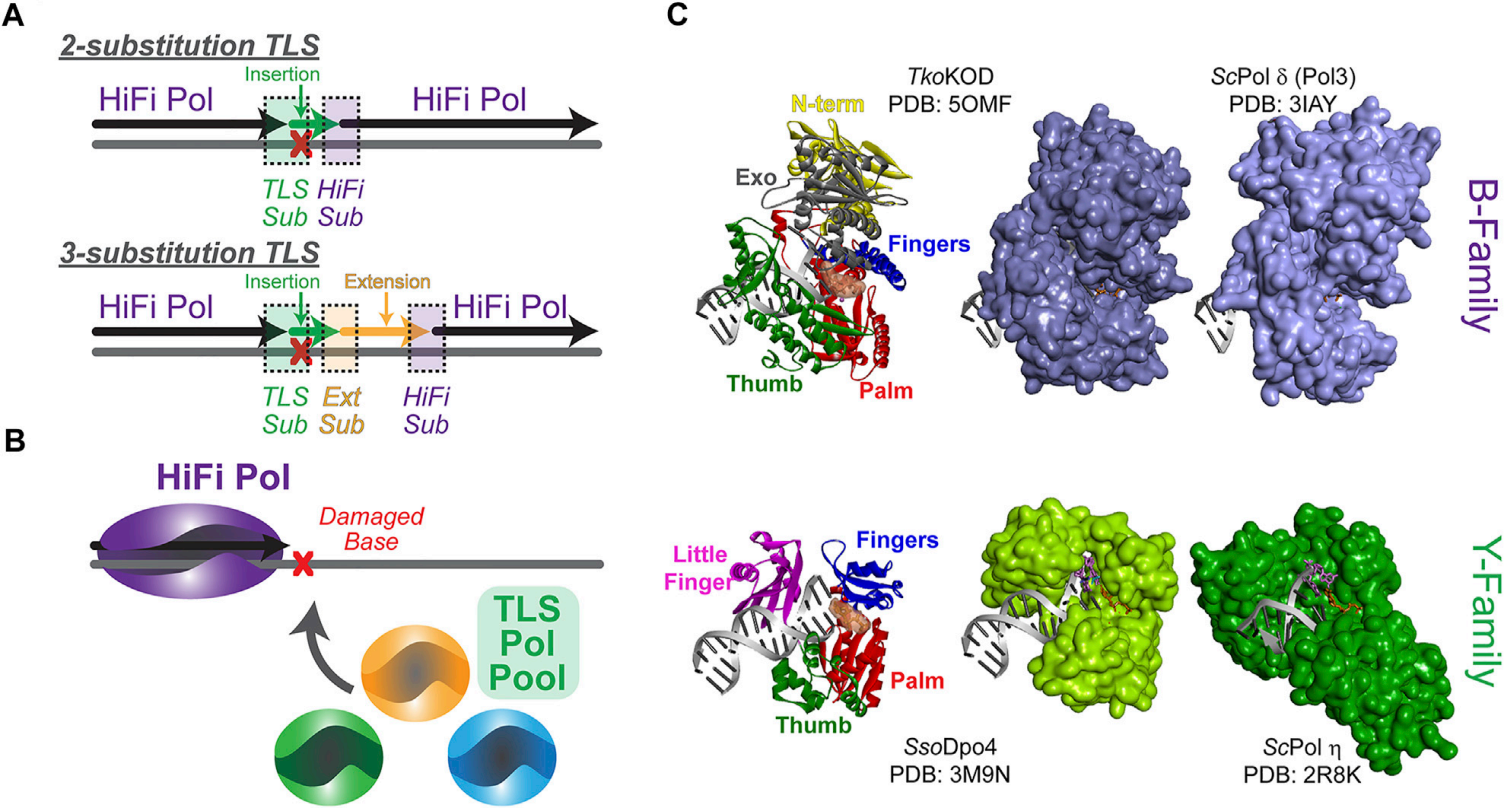
Steps required for the overall translesion synthesis (TLS) process. **(A)** A high fidelity (HiFi) polymerase (Pol) will stall at a lesion requiring two- or three-substitution TLS for bypass. In a two-substitution mechanism, the first TLS substitution (sub) will recruit an inserter TLS Pol (green) for translesion DNA insertion (green) followed by substitution back to a HiFi Pol (purple). In a three-substitution mechanism, an extender (Ext) TLS Pol is substituted after insertion and is required for limited downstream extension (orange) prior to substitution back to the HiFi Pol. **(B)** TLS substitution will be influenced by the available TLS Pol Pool as well as the specificity for a particular lesion. **(C)** Compares the polymerase domain structure (N-term—yellow, Exo—grey, Palm—red, Fingers—blue, Thumb—green, and Little Finger—pink) for the more closed structures of HiFi B-family Pols KOD (*Thermococcus kodakarensis*) (Kropp et al., 2017) and Pol δ (*Saccharomyces cerevisiae*) (Swan et al., 2009) with the more open active sites of TLS Y-family Pols *Sso*Dpo4 (Wong et al., 2010) and Pol η (*Saccharomyces cerevisiae*) (Alt et al., 2007) in complex with DNA, all arranged in the same orientation.

DNA damage can come from a variety of endogenous and exogenous sources and persist within the genome into the synthesis (S) phase of the cell cycle (Hakem, 2008). During S-phase, high fidelity (HiFi) polymerases (Pols) duplicate complementary DNA strands with a rapid rate of synthesis and a low rate of error. However, upon encountering DNA base damage within a template strand, HiFi Pols are unable to continue DNA synthesis and are stalled in place. Specialized Pols capable of bypassing the template-strand lesion, known as translesion synthesis (TLS) Pols, are recruited to the site of the damage, substituted in for the HiFi Pol, and then used to synthesize over and past the lesion (Figure 1A). TLS Pols have evolved to ensure error-free synthesis across preferred lesions but have overall lower fidelity than HiFi Pols (Waters et al., 2009; Goodman and Woodgate, 2013; Sale, 2013; Fujii and Fuchs, 2020). Following successful insertion of one or more nucleotides opposite (or past) the lesion, TLS Pols must dissociate from the DNA template and substitute back to the replicative Pol to resume high fidelity genomic synthesis (Trakselis et al., 2017).

Although relatively simple in description, there are many intricate elements of the TLS mechanism. Previously, TLS has been described by a “one-polymerase” or “two-polymerase” process; however, the number and subunit composition of Pols found at TLS sites is expanding, and while some Pols have direct synthesis activities, others act only as scaffolds for assemblies. Therefore, the diversity of TLS processes would be better defined by a “two-substitution” or “three-substitution” mechanism (Figure 1A), focusing primarily on the steps required for synthesis instead of individual Pols involved. Insertion opposite a lesion requires that an appropriately selected TLS “inserter” Pol is substituted for the HiFi Pol. After TLS insertion, there can be direct substitution back to the HiFi Pol to resume synthesis (i.e., two-substitution) or subsequently an intermediary “extender” Pol can synthesize downstream of the lesion prior to substitution back to the HiFi Pol in a three-substitution mechanism. There are several mechanistic facets that regulate this process to help select TLS Pols from the cellular pool (Figure 1B) and restrict their activities after TLS for the benefit of maintaining a stable genome. Although many of these basic principles have been better characterized in prokaryotic systems, where a more limited set of Pols are available, this review will focus primarily on defining the TLS process in more complex eukaryotic systems, culminating with mechanistic hypotheses for limiting lower fidelity TLS Pol synthesis downstream of the lesion to favor resumption of high-fidelity synthesis.

### Translesion Synthesis Pol Open Active Site

All DNA Pols resemble a right hand, with a structure consisting minimally of palm, finger, and thumb domains, comprising the catalytic core (Figure 1C). This conformation allows for binding of the DNA substrate while simultaneously inserting nucleotides during DNA synthesis. HiFi Pols also include a second exonuclease (Exo) active site that is utilized to proofread insertions and ensure fidelity. While HiFi Pols of the B-family have smaller and more closed active sites to ensure efficient and accurate DNA replication opposite undamaged template strands, TLS Pols of the Y-family have larger and more open active sites that allow insertion of one or more nucleotides opposite a lesion. Y-family TLS Pols generally have small finger domains and have an additional little finger (LF) domain that attenuates lesion bypass abilities and processivities (Boudsocq et al., 2004; Pata, 2010). Varying distances between the LF and the catalytic core, creating a structural gap, add specificity to the size of lesions that each TLS Pol can bypass (Yang and Gao, 2018). As a result of these structural differences, TLS Pols are capable “inserters” opposite specific lesions but are inherently less accurate and can quickly generate errors in the nascent DNA strand when synthesizing opposite undamaged DNA (Vaisman and Woodgate, 2017). Although these Pols have tolerant active sites to accommodate bulky lesions, mutagenesis even during TLS is fairly common (Volkova et al., 2020). Therefore, it is paramount to cellular survival that synthesis by certain TLS Pols be restricted only to base insertion opposite DNA lesions to prevent misincorporations downstream. HiFi Pols with more discriminative active sites must promptly resume replicating DNA to minimize inaccurate synthesis. To facilitate the end of a TLS event, the DNA replisome needs to *exchange* or *switch* back to the HiFi Pol to proceed with faithful DNA replication.

### Translesion Synthesis Pols and Lesion Specificity

DNA damage can lead to genomic instability and carcinogenesis (Hoeijmakers, 2009; Shilkin et al., 2020). TLS serves as a DNA damage tolerance mechanism by allowing DNA replication to persevere in the presence of DNA lesions. By allowing DNA synthesis to continue upon the encountering of a template-strand lesion, TLS Pols reduce the risk of cellular apoptosis and prevent occurrence of double-strand breaks from excessive replisome stalling (Chatterjee and Walker, 2017).

Before discussing the plethora of Pols capable of performing TLS, it is important to understand the types of lesions that are encountered as well as their sources. For DNA-damaging agents that have been prevalent for long periods of time, it is possible that TLS Pols have evolved to specifically bypass the resulting lesions (Livneh et al., 2010). For example, eukaryotic TLS Pol η is capable of perfectly bypassing thymine cyclobutane pyrimidine dimers (CPDs) following UV radiation from sunlight with correctly base-paired adenines (Johnson et al., 2000b; Biertumpfel et al., 2010) (Figure 1C). Separately, archaeal DNA polymerase 4 (Dpo4) can synthesize nascent DNA beyond 7,8-dihydro-8-oxoguanine (8-oxoG) lesions generated by reactive oxygen species, as well as many additional lesions (Yang, 2014; Cranford et al., 2020; Jung and Lee, 2020) (Figure 1C).

Relatively newer sources of exogenous damage, such as platinating agents used in cancer treatment, have not yielded an evolutionary adaptation in a specific TLS Pol and are instead bypassed by the most suitable (or a combination of multiple) Pol(s). In organisms with multiple available TLS Pols, specificity to lesions is a determining factor for which Pol will perform the bypass (Yang, 2014). Human cells contain four Y-family TLS Pols: Pols η, ι, κ, and Rev1, each with varying active site dimensions and unique bypass capabilities. Pols η, ι, and κ have all been shown to act as the “inserter” Pol opposite a variety of lesions, with the large active site of Pol κ allowing bypass of the bulkiest lesions (Vaisman et al., 2001; Kusumoto et al., 2002; Albertella et al., 2005a; Alt et al., 2007; Yoon et al., 2009; Phi et al., 2019; Jung et al., 2020; Vaisman and Woodgate, 2020; Ghodke et al., 2021). Human Pol ν, an A-family Pol, has also demonstrated an ability to act as an “inserter” TLS Pol opposite lesions (Takata et al., 2006; Gowda and Spratt, 2016; Du et al., 2020). While structurally similar to other Y-family Pols, Rev1 acts less frequently as an “inserter” and more often as a scaffold to facilitate the binding of other Pols through conserved Rev1-interacting regions (RIRs) (Qin et al., 2013; Boehm et al., 2016b; Pustovalova et al., 2016). Recent studies have shown that Pol λ, an X-family Pol, can also perform this scaffolding role during TLS (Yoon et al., 2021). The varying active site structures, activities, and potential contacts of TLS Pols regulate Pol selection for lesion bypass.

## Polymerase Holoenzyme Complexes With PCNA

A polymerase holoenzyme complex is assembled to provide some stability when bound to DNA that presents biochemically as processivity. Processivity can be defined by an equation that quantifies the probability of a Pol continuing at a specific site based on the *k*_*pol*_ for nucleotide incorporation relative to *k*_*off*_ for dissociation from the DNA template (Hippel et al., 1994). Several mechanisms have evolved to increase processivities of HiFi Pols and limit processivities of TLS Pols to ensure genome stability. For Pols, the trimeric clamp protein (proliferating cell nuclear antigen, PCNA, in archaea and eukaryotes) encircles duplex DNA and provides a topological link to the DNA, effectively reducing *k*_*off*_ (Trakselis and Benkovic, 2001; Boehm et al., 2016a).

The binding affinity of the Pol itself for DNA can also impact the processivity. Interestingly, HiFi Pols generally have a higher affinity for PCNA than DNA, while TLS Pols have a higher affinity for DNA than for PCNA (Lin et al., 2012; Hedglin et al., 2016a). This implies that binding to clamp proteins is often required for high fidelity and processive DNA synthesis, while TLS activity may be more restricted directly by the DNA lesion. This provides a plausible mechanism for limiting TLS, however, the mechanism for releasing a TLS Pol from DNA after translesion synthesis is not well understood.

**FIGURE 2:**
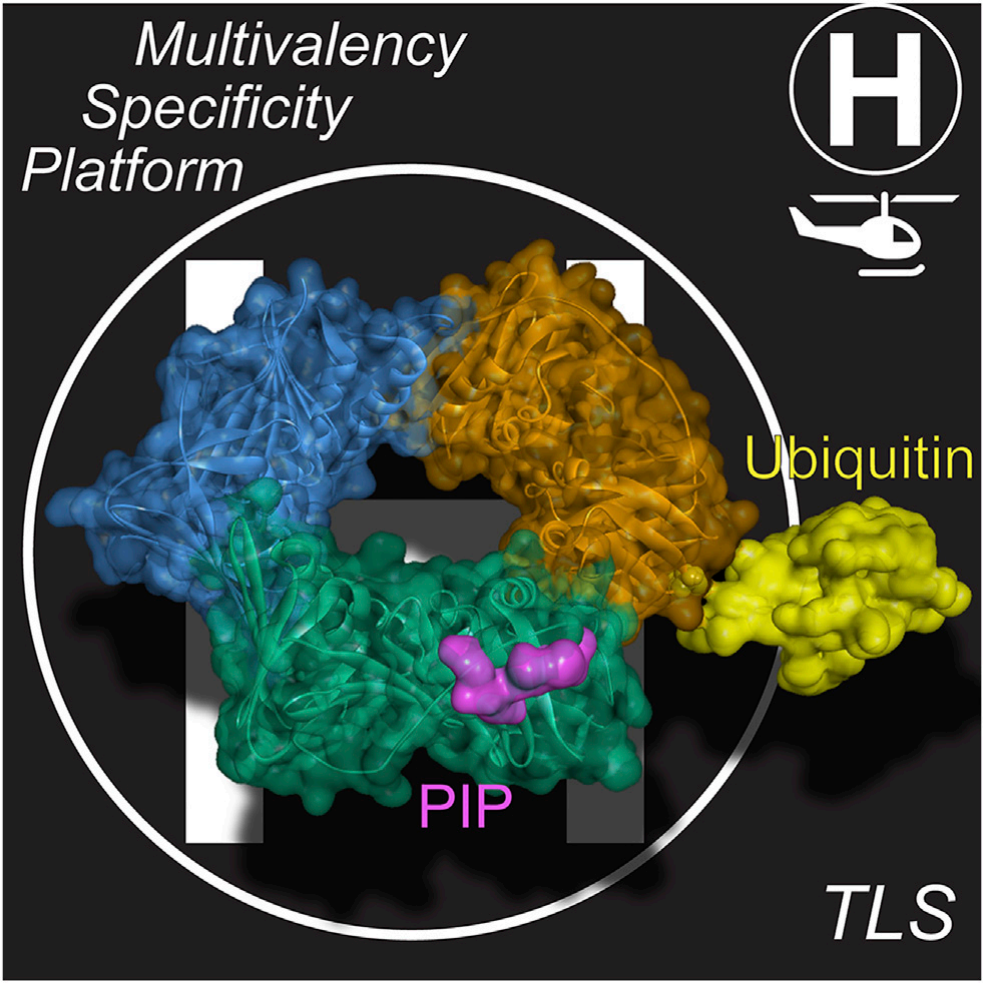
PCNA is a dynamic “landing-pad” (or platform). PIP binding sites at the interdomain connecting loop (IDL) and ubiquitination at K164 are available on each of the three PCNA subunits, providing for multivalency. Structural model derived from PCNA/p21 peptide structure (PDBID: 1AXC) (Gulbis et al., 1996) and monoubiquitinated PCNA structure (PDBID: 3TBL) (Zhang et al., 2012).

Pols have several interaction sites with the clamp, but in eukaryotes and archaea, the primary interaction site is through a hydrophobic PCNA-Interacting Protein (PIP) patch, adjacent to the interdomain connecting loop within a single PCNA monomer (Figure 2). This PIP binding site is located on the front (or leading face) of PCNA and interacts with proteins through a specific motif in a partner protein containing the general consensus sequence, *Qxxhxxaa*, where *h* is hydrophobic and *a* is aromatic (Warbrick, 1998). Glutamine binds to a Q-pocket through specific hydrogen bonding contacts, while the aromatic residues lay within the hydrophobic pocket. Although more than 75 proteins, including several DNA repair proteins and cell cycle regulators, contain this consensus PIP motif, TLS Pols generally encode a nonconsensus PIP motif, (K/G)xx(I/L)xx(FY/L)(FY/L) that bypasses the Q-pocket (Slade, 2018; Dash and Hadden, 2021). One to three PIP motifs are located in the unstructured C-terminal extensions of Y-family Pols (Powers and Washington, 2018). The variability within the consensus and nonconsensus PIP motifs can alter the *K*_*d*_ of interaction widely from 10 nM to >30 µM (Slade, 2018; Prestel et al., 2019).

It is well known that upon extreme stalling, PCNA becomes monoubiquitinated (mUB) at K164 (Figure 2) (Stelter and Ulrich, 2003; Kannouche et al., 2004; Chang et al., 2006). This additional ubiquitin facet on PCNA can further increase the binding affinity of TLS Pols though a ubiquitin-binding zinc finger (UBZ) motif (Bomar et al., 2007; Pillaire et al., 2014) or a ubiquitin-binding motif (UBM) (McIntyre et al., 2015; Vanarotti et al., 2018) also within the unstructured C-terminal tails of Y-family TLS Pols (Yang and Gao, 2018). mUb-PCNA acts to not only recruit TLS Pols at stalled replication forks but also to increase their residence times to carry out TLS (Sabbioneda et al., 2008; Sabbioneda et al., 2009). The covalent addition of Ub to the back side of PCNA^K164^ and the flexibility inherent within the unstructured C-terminal tails containing these motifs allow these TLS Pols to adopt several conformations on PCNA in inactive carrier or active polymerizing states (Shen et al., 2021). Therefore, based on this multivalency for trimeric mUb-PCNA, there are now at least six specific contact points for recruiting and interacting with Pols, providing a landing platform for assemblies (Figure 2) in what has been described previously as a “tool-belt” model (Kath et al., 2014; Boehm et al., 2016b; Cranford et al., 2017). A holoenzyme complex, consisting of the synthesizing Pol bound to PCNA, or a supraholoenzyme complex, comprised of multiple Pols bound to PCNA, contain many protein-protein interactions (PPIs) for added stability during DNA synthesis.

## Mechanisms for the Translesion Synthesis Switch or Exchange

**FIGURE 3:**
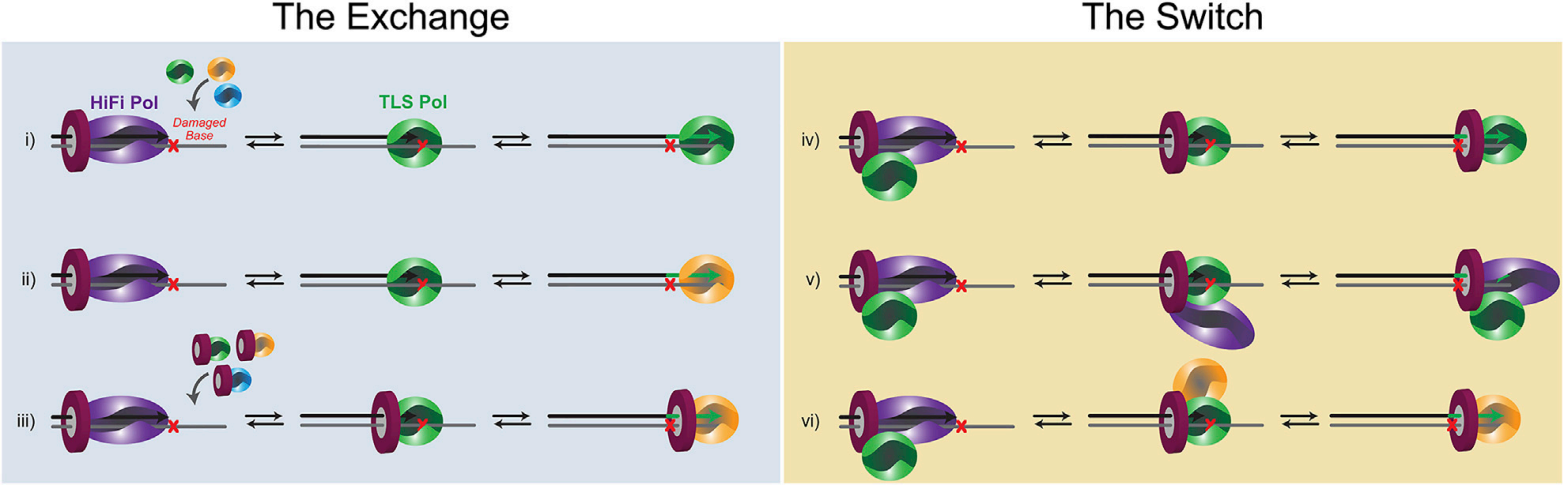
Schematic of the potential equilibrium *exchanges* and PCNA-directed *switches* that occur for TLS. **(i)** a distributive Pol exchange before two-substitution TLS. **(ii)** A distributive Pol *exchange* before three-substitution TLSs.**(iii)** a distributive PCNA/Pol *exchange* before two (or three)-substitution TLS. **(iv)** A concerted Pol *switch* with a pre-associated TLS Pol. **(v)** A concerted Pol *switch* with the HiFi Pol remaining bound to PCNA throughout TLS. **(vi)** Concerted Pol *switches* during three-substitution TLS.

Substitution of a HiFi Pol for a TLS Pol to hand off the primer opposite a damaged template is required to insert opposite a variety of template lesions, however, the exact mechanism for the polymerase *exchange* or *switch* is uncharacterized. Moreover, the diversity of lesion types and abundance of Pols within the cell would not suggest that a single mechanism is utilized. Instead, it is likely that several mechanisms are employed dependent on the specific lesion, the DNA context, and the signaling pathways. Conceptually, substitution of one Pol for another that relies primarily on kinetic modulation (i.e., *k*_*off*_) can be considered equilibrium *exchange* events, while those that are mediated through PPIs directly between Pols or through PCNA in a “tool-belt” or “landing-pad” fashion are *switches* (Figure 3). It is probable that aspects of both of these basic mechanisms are employed. For example, recruitment of a TLS Pol to a stalled HiFi holoenzyme may be brought in by specific contacts with PCNA (Figure 2) or combined with Pol-Pol interactions creating a transient supraholoenzyme to initiate a *switch* only to then have the HiFi Pol and/or PCNA dissociate in a subsequent *exchange* for insertion opposite a lesion.

### A Distributive Mechanism of Polymerase “Exchange”

Stalling of a DNA Pol at a lesion or template-blocking event will render it catalytically inactive. In that case, *k*_*pol*_ becomes extremely slow, and the *k*_*off*_ event will predominate, dissociating the Pol from DNA. *k*_*off*_ can be increased further by minor DNA distortions caused by the lesion itself (Broyde et al., 2008) or from shuttling between polymerase and exonuclease active sites within the HiFi Pol (Khare and Eckert, 2002). In this situation, multiequilibrium processes will predominate, and depending on the affinity of an exchanged Pol for the lesion and the concentration of Pols within the cell, a new TLS Pol would be selected (Figure 3). In the *exchange* model, very little, if any, contacts are retained between an incoming TLS Pol and either the HiFi Pol or PCNA. Instead, the template becomes accessible after a *k*_*off*_ event, and a new TLS Pol can bind the damaged template for an insertion event. After insertion, the same TLS Pol can extend for several bases past the lesion, dependent on its own processivity and synthesis properties. Alternatively, another Pol substitution event can be utilized to synthesize past the lesion functioning as an “extender” Pol. Thus, the “hand-off” is indirect and is more consistent with an external equilibrium Pol active site *exchange* of the primer/template.

### A Concerted Polymerase ‘Switching’ Mechanism

There is also evidence for several PPIs or contacts within a Pol holoenzyme. This occurs primarily through multivalency of binding to the trimeric PCNA clamp but can also be through direct Pol-Pol interactions. Several Pol interactions have been discovered between B- and Y-family Pols (designated YB sites) (Baldeck et al., 2015; Cranford et al., 2017). The key to a successful *switch* is that enzyme dissociation-association events from DNA are restricted in favor of pre-bound Pols releasing and swapping active-site binding to the damaged template (Figure 3). The affinity of TLS Pols for PCNA or the HiFi Pol itself effectively increases the local concentrations of TLS Pols at the replication fork, allowing for a more coordinated *switch*. To be clear, there can be several *k*_*off*_ events involved in a switch. The first, and more traditional event, would be *k*_*off-DNA*_ of the HiFi Pol from DNA, but in that case, localized binding can be retained by PCNA. Should the HiFi Pol dissociate from PCNA, a second *k*_*off-PCNA*_ event can occur. Of course, further contacts such as *k*_*off-Pol*_ between Pols (i.e., YB site) can also help mediate a *switching* event. In support, mutation of the YB site in the archaeal *Saccharalobus solfataricus* (*Sso*)Dpo4 enzyme results in decreased processivity of DNA synthesis suggesting that the supraholoenzyme complex is a more stable synthesizer (Cranford et al., 2017). Upon successful *switching*, the fate of the HiFi Pol is not always clear. It can dissociate from PCNA and the DNA template and be lost to solution, or it can be held in check within the supraholoenzyme complex. In a TLS *switch*, the “hand-off” is direct with contacts between Pols or coordinated through multivalent PCNA contacts facilitating primer/template binding to a preassociated Pol.

The kinetics of the entire TLS process from stall, substitution, insertion, and extension will be influential in determining whether an *exchange* or *switch* is more likely. For example, weaker PPI contacts and slower rates of insertion opposite more difficult lesions may favor a more distributive *exchange* of Pols from solution. However, stronger interactions and faster insertion kinetics may allow a concerted *switch* to be more likely, through the confines of a supraholoenzyme complex. Therefore, the PPI affinities, the equilibria concentrations of Pols, and the catalytic efficiency of an insertion (dependent on the lesion type) will be linked, making this highly dynamic process difficult to measure and accurately characterize.

### Limitations for Biochemical Assays Designed to Examine Polymerase Substitutions

**FIGURE 4:**
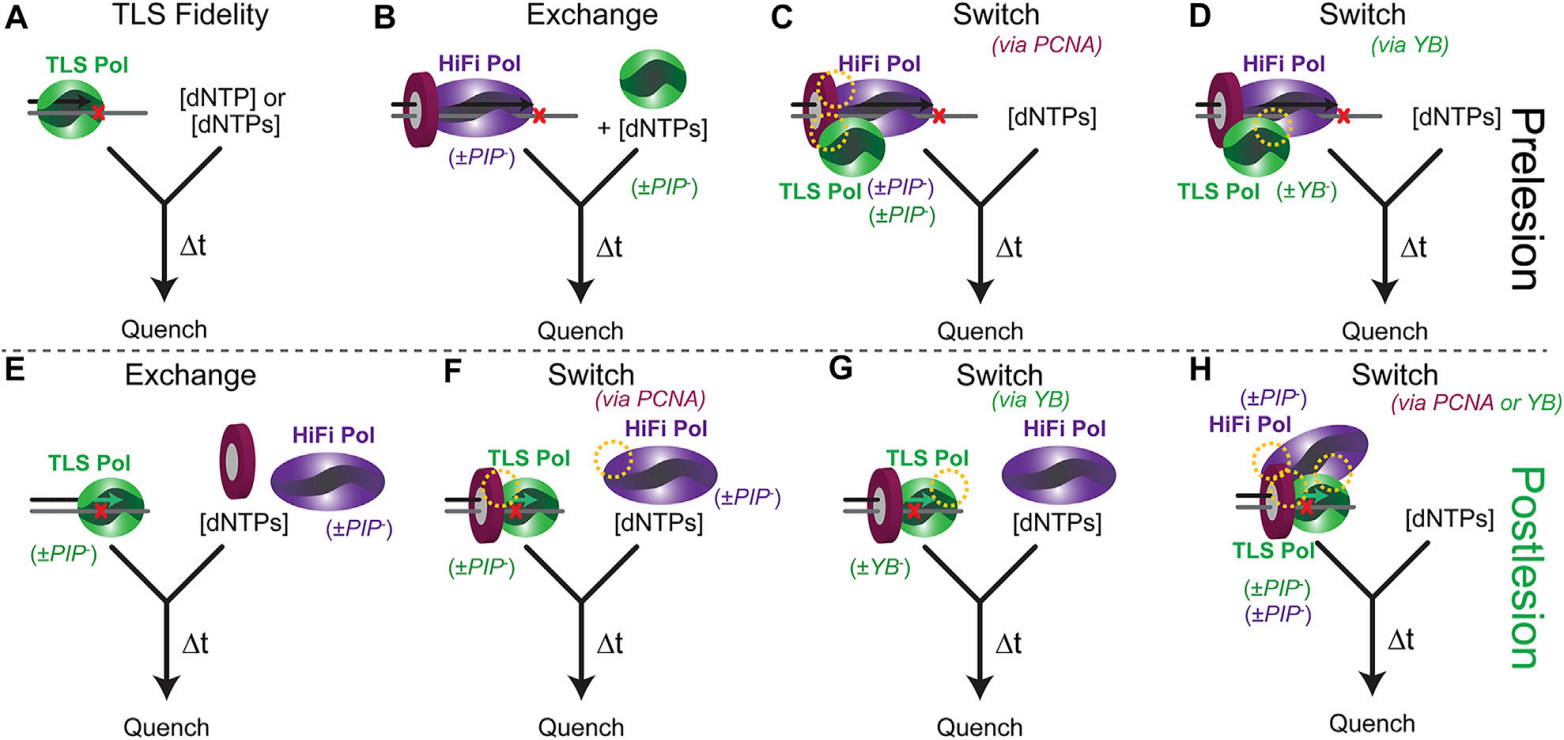
Experimental presteady-state schemes to examine the TLS process both pre and post lesion. **(A)** TLS Pol and various DNA template lesions to examine inherent fidelity in isolation. **(B)** Prebound HiFi Pol with PCNA stalled to examine a kinetic *exchange* process. Preassembled supraholoenzyme complex with HiFi and TLS Pols bound to DNA to examine kinetics of *switching* directed **(C)** by interactions with PCNA (PIP sites) or **(D)** through Pol-Pol interactions (YB site) (yellow circles). **(E)** A TLS Pol with a primer that is a defined number of bases past a lesion is used to examine a distributive *exchange*, **(F)** a PCNA directed *switch*, or **(G)** a YB directed *switch*. **(H)** The kinetics of a preassembled PCNA/TLS/HiFi Pol directed *switch* back to high fidelity synthesis can be validated by utilizing PIP^−^ or YB^−^ mutants of all Pols.

There are several inherent complexities for a biochemical experiment to fully examine Pol substitutions preceding, during, or after a TLS event. Typically, an *in vitro* dynamic biochemical kinetic characterization can be performed with only a limited and feasible number of biological components in a single experiment. Countless kinetic fidelity measurements have been made for many DNA Pols for even more DNA lesions in a single enzyme—single substrate experiment (Figure 4A), providing valuable quantitative understanding of lesion bypass and fidelity (Berdis, 2009; Liu et al., 2016; Powers and Washington, 2017; Raper et al., 2018). As the overall TLS process can include several proteins, numerous PPIs, differing kinetics, and variable equilibria, this experimental setup can be complicated. It is common to mutate the exonuclease proofreading site of the HiFi Pol to eliminate the degradation of the primer strand that complicates the extension kinetics, but the shuttling between Pol and Exo sites may be influential in directing the TLS process (Reha-Krantz, 2010). Moreover, it is also common to truncate TLS Pols to their core polymerase domain, removing important PIP/RIR/UBM/UBZ motifs in the unstructured C-terminal tails and making the examination of *switching* impossible.

The presteady-state kinetics of TLS insertions and extensions can be monitored to better understand the impact of various *exchange* or *switch* processes using a rapid quench setup (Figures 4B–D). The experimental design is important as it impacts the desired measured outcome. For example, the *k*_*on*_ for a TLS pol is usually diffusion-limited and rapid. However, if that is not the case then the method used to examine an exchange (Figure 4B) may be affected. Addition of a TLS Pol after a stalled HiFi Pol will simulate an incoming (or recruited) TLS Pol. The TLS Pol concentration can be varied to examine critical aspects of competitive binding *exchange*. Preincubation of HiFi and TLS Pols with PCNA and DNA can simulate and test a “tool-belt” model of concerted Pol *switching*. The kinetics of insertion and extension may vary depending on whether the TLS Pol is included initially or subsequently to help validate one particular model. The DNA primer length can also be altered to examine any kinetic differences of an approaching DNA Pol holoenzyme with a pre-stalled one in a “running start” assay. Catalytically deficient mutations (*cat* ^*−*^) in either the HiFi or TLS Pol active sites can be used to distinguish extension products when multiple Pols are included. PPIs can be disrupted through site-specific mutations of the PIP (*PIP* ^*−*^) or YB (*YB* ^*−*^) sites for each Pol to test the impact of these contacts in facilitating a TLS *switch*. To fully understand the implications of Pol interactions with PCNA in a *switch*, full length, and not truncated core forms, of TLS Pols should be utilized.

After insertion, the stability and processivity of the TLS Pol can be examined. The design of these experiments will again test whether there is a second distributive *exchange* or facilitated *switch* to resume processive synthesis (Figures 4E–H). The DNA primer is systematically lengthened to determine whether there is a position of inherent destabilization of the TLS Pol that limits further extension. PCNA can be excluded or included with the TLS Pol (+/− Rev1) to initiate extension with mixing the HiFi Pol. The resulting product distributions and maximal kinetics of extension will inform on the preferred scheme. For eukaryotic systems, Ub can be covalently added to PCNA through several chemical biology approaches (Freudenthal et al., 2010; Hibbert and Sixma, 2012; Yang et al., 2016; Gong et al., 2018). Combined with UBM/UBZ mutations in the C-termini of TLS Pols, the role for mUb-PCNA in the TLS process can also be explored through the kinetics of extension.

## Prelesion: Stalling of the High Fidelity Polymerase

Upon encountering a template lesion or a difficult to replicate region from secondary structure or physical impediments, the HiFi Pol will stall (Marians, 2018; Maiorano et al., 2021). The HiFi Pol will attempt insertion of a nucleotide opposite the lesion, however it is almost always unsuccessful (McCulloch and Kunkel, 2008). If insertion opposite a lesion is successful, then further extension is severely inhibited because of distortion in the duplex that is sensed, and the terminal nucleotide is removed at the exonuclease proofreading active site of the HiFi Pol. This stalling (or shuttling between active sites) will set in motion a series of events that act to either stabilize the replication fork for downstream repair or initiate TLS to rapidly continue (Aguilera and Gomez-Gonzalez, 2008; Marechal and Zou, 2013).

After stalling, the HiFi Pol will decouple from the replication helicase causing a buildup of single-strand DNA (ssDNA) (Byun et al., 2005; Chang et al., 2006; Guilliam and Yeeles, 2020). ssDNA alone is fragile and must be protected from breakage by RPA or Rad51 to prevent loss of genetic material and can be a checkpoint signal for repair through the ATR kinase signaling pathway (Cimprich and Cortez, 2008; Blackford and Jackson, 2017; Lanz et al., 2019). Transient stalls, where no direct DNA damage is present, may be relieved by a host of fork stabilizers, accessory factors, and restart proteins that can remove a physical block and allow synthesis and coupling to resume (Lopes et al., 2006; Rickman and Smogorzewska, 2019; Qiu et al., 2021). It has even been shown that the eukaryotic lagging strand Pol δ can efficiently substitute for a decoupled leading strand Pol ε when smaller lesions such as 8-oxoG or thymine glycol are present (Guilliam and Yeeles, 2021). However, if a template lesion is more severe and unsurpassable by the HiFi Pol, then other TLS Pols will be recruited to attempt bypass (Ma et al., 2020).

## Prelesion: The First Translesion Synthesis Polymerase Substitution

After stalling of the HiFi Pol at a lesion, it must dissociate from the primer/template DNA through one of the above substitution mechanisms (Figure 3). The replicative Pol will either fully dissociate from the primer-template junction into solution, following a distributive *exchange* mechanism, or it will remain bound to PCNA in accordance with a concerted *switching* mechanism. For both the archaeal Pol B1 and eukaryotic Pol δ HiFi Pol holoenzyme systems, it was found that the HiFi Pol readily dissociates in a distributive exchange mechanism even during normal DNA synthesis (Bauer et al., 2013; Hedglin et al., 2016b). The lagging strand HiFi Pol in humans, Pol δ, has PIP-sites on three of its four subunits, however only mutation of the PIP-site in the large catalytic subunit significantly reduces Pol activity (Lancey et al., 2020). This potentiates multiple Pols being bound to other PCNA subunits simultaneously in a supraholoenzyme, or at least that multiple PCNA subunits can facilitate *exchange* of Pols (as a landing pad) during TLS. Aside from specific contacts with PCNA, there are several other factors regulating TLS Pol recruitment and selection prior to lesion bypass.

### Translesion Synthesis Pol Recruitment and Selection

In mammalian cells, Pol δ is known to convert from a four-subunit to a three-subunit enzyme complex both upon S-phase entry (Zhang et al., 2013) and specifically in response to DNA damage (Zhang et al., 2007; Lee et al., 2014a). Degradation of the smallest subunit, p12, serves as a regulatory mechanism to activate Pol δ for synthesis normally and is further directed by ATR signaling (Zhang et al., 2007; Lee et al., 2019). From here, TLS Pol recruitment and selection can depend on a multitude of factors, including the aforementioned PIP or UBZ/UBM interactions with (Ub-)PCNA (Figure 2), Pol affinity for the damaged DNA substrate, and Pol-Pol interactions. Various types of DNA-damaging conditions have been shown to localize TLS Pols in replication foci in nuclei, indicating that Pol concentration is an early contributor of selection for TLS (Maiorano et al., 2021). Once TLS Pols are in the vicinity of the damage site, Pol affinity for the DNA substrate becomes a driver of Pol selection. Accommodation of lesions by TLS Pols can be influenced by template-strand sequence and steric hindrance between bulkier lesions and Pol active sites (Zhao and Washington, 2017; Thomforde et al., 2021). Sequence context has also been shown to affect Pol efficiency and accuracy during TLS opposite various lesions, however the molecular basis for this effect is not well understood (Shriber et al., 2015; Bacurio et al., 2021).

Despite these qualifications for TLS by certain Pols to occur, it should be noted that backup TLS pathways exist in eukaryotes. Certain lesions deter recruitment of some TLS Pols in favor of more suitable inserters, however in the absence of the favored Pol, a less-favored TLS Pol(s) can bypass these lesions. Secondary TLS mechanisms have been demonstrated both *in vivo*, through Pol knockdowns, and *in vitro*, by examining multiple TLS Pol bypass capabilities opposite an assortment of lesions (Yoon et al., 2009; Livneh et al., 2010; Jha and Ling, 2018; Inomata et al., 2021). The presence of backup TLS pathways indicates that steric effects may not fully regulate Pol selection, but likely contribute significantly.

## Postlesion: Extension Past the Lesion, a Possible Second Substitution

After insertion of a base opposite a lesion, the TLS Pol can continue synthesizing and extending downstream until inherent enzymatic properties, PPIs, or multiequilibria processes allow for substitution of other Pols. TLS Pols have no exonuclease proofreading domains and lower fidelities compared to HiFi Pols (Figure 1C), and so, their lower processivities serve a vital function to limit further DNA synthesis past a lesion to maintain genome integrity. However, the mechanisms for extension and reestablishing the HiFi holoenzyme past a TLS event are not well studied.

### Translesion Synthesis Polymerases: From Inserters to Extenders

Beyond the lesion, “inserter” TLS Pols extend the nascent DNA strand to a position where either an “extender” TLS Pol, such as eukaryotic Pol ζ, can continue TLS or where the HiFi Pol can resume synthesis. A TLS mechanism in which an “inserter” Pol is the only Pol required for TLS can be termed “two-substitution” TLS. In the event both an “inserter” and “extender” Pol is required for TLS, the term “three-substitution” TLS can be used. The vocabulary of “substitution” is preferred over other vague describers including “one-“ or “two-polymerase” TLS (Johnson et al., 2000a) to focus more on the mechanistic process. With Rev1 potentially being involved as a scaffold in both two- and three-substitution mechanisms, defining the TLS by the number of Pols may be inaccurate and confusing. Therefore, nomenclature referring to the number of “substitutions” is preferred. Pol ζ has been shown to function as the primary “extender” Pol after the “inserter” in a “three-substitution” TLS process for certain lesions (Shachar et al., 2009; Lee Y.-S. et al., 2014). A-family Pol θ has also displayed some “extender” activity, although its exact role is still not fully defined (Seki et al., 2004; Bacurio et al., 2021). Recently, *Sulfolobus islandicus* B-family polymerase Dpo2 has been shown to act as the “extender” Pol in combination with “inserter” Pol Dpo4 during bypass of abasic sites (Feng et al., 2021).

### Pol ζ Recruitment and Assembly

Assembly of the five-subunit extender Pol ζ is facilitated by HiFi Pol δ and the Y-family Rev1. Although the precise timing of this mechanism is not clear, Pol δ can rearrange and share subunits with Pol ζ to form the active Pol ζ complex. Pol δ shares subunits Pol31 and Pol32 in yeast and subunits p50 (Polδ2) and p66 (Polδ3) in humans with Pol ζ (Baranovskiy et al., 2012; Malik et al., 2020). Aside from the shared subunits, Pol ζ is comprised of Rev3, the catalytic subunit, simultaneously bound to two Rev7 subunits (Rizzo et al., 2018). The entire Pol ζ complex interacts in a 1:1 ratio with the scaffold TLS Pol Rev1. Rev1 is presumably bound to the replisome during an initial response to DNA damage and is soon thereafter able to recruit Pol ζ to the primer/template junction, coordinating the inserter-to-extender three-substitution mechanism (Makarova and Burgers, 2015; Liu et al., 2018). In a two-substitution mechanism, such as Pol η bypass of a UV-induced CPD, there is no requirement for an extender Pol. Under such circumstances, Pol ζ recruitment and assembly would likely not occur, even though the initial DNA damage response may generate mUb-PCNA and recruit Rev1. Interestingly, when Pol η is absent, Pol ζ functions in subsequence to Pol ι or Pol κ in TLS opposite this exact lesion (Ziv et al., 2009). The ability of the extender Pol ζ to function in a backup TLS mechanism when it is not required in the primary bypass mechanism for a certain lesion indicates that Pol ζ recruitment and assembly may always occur in response to DNA damage. Another possibility is that Pol ζ assembly does not occur until after the “inserter” Pol has performed the first step of TLS, at which time synthesis is stalled until a suitable “extender” can be recruited to the DNA substrate. Further experimentation needs to be performed to ascertain the exact timing of Pol ζ recruitment and assembly.

## Postlesion: Resuming High Fidelity Synthesis, a Final Substitution

**FIGURE 5:**
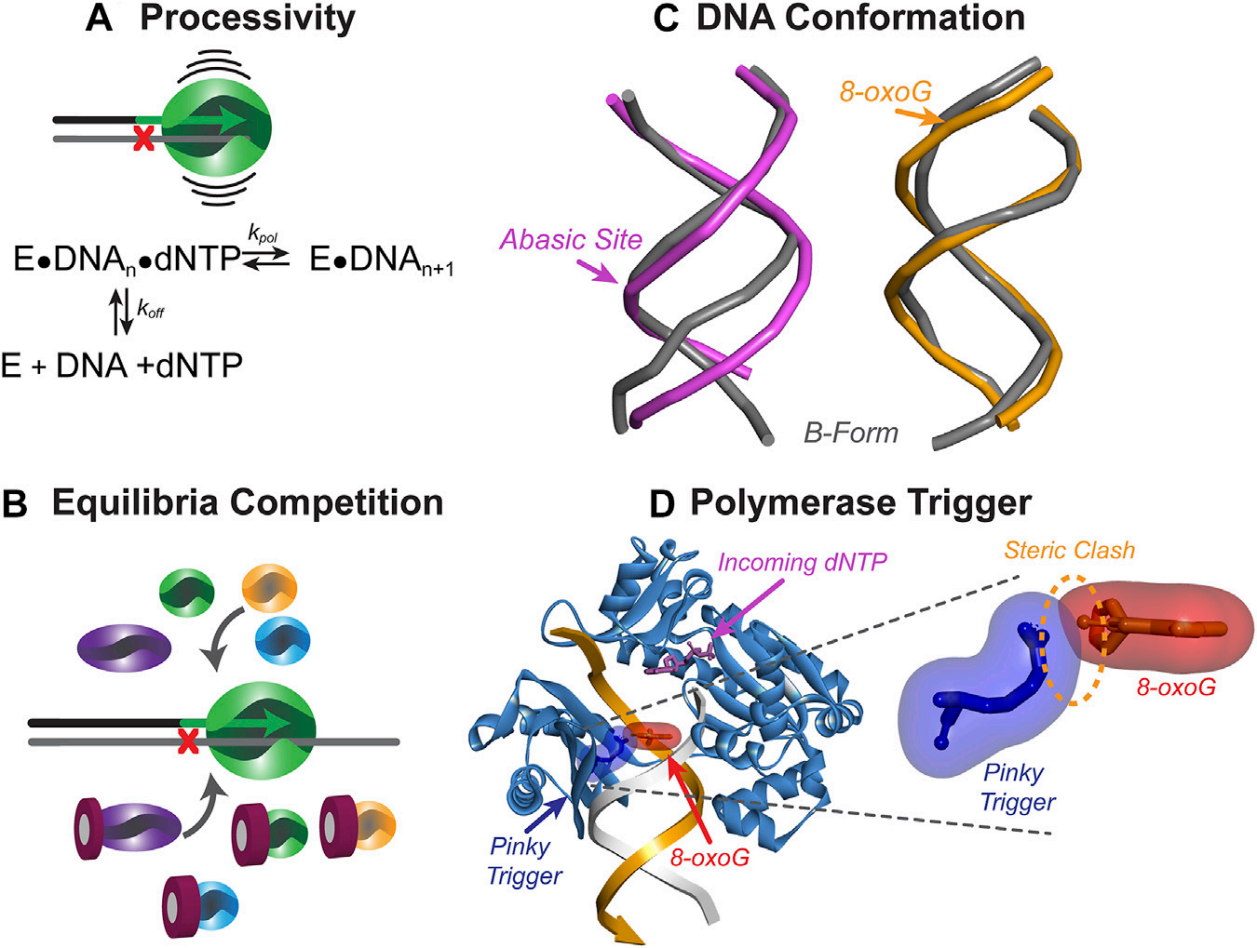
Mechanisms to initiate resumption of high-fidelity synthesis. **(A)** The processivity of a Pol represents the number of nucleotides inserted before dissociating from DNA and calculated as a ratio of *k*_*pol*_ to *k*_*off*_. TLS Pols have lower processivity than HiFi Pols and can readily dissociate from DNA when the bypassed lesion is no longer in the active site. **(B)** DNA-damaging events can cause upregulation of TLS Pols, leading to concentration-dependent competition between Pols in solution for access to the DNA substrate. **(C)** DNA lesions can alter the conformation of the DNA, making extension of the nascent strand more difficult for the synthesizing Pol. Abasic site duplex (pink, PDB: 2HSS (Chen et al., 2007)) show more distortion than 8-oxo-G duplex (orange, PDB: 5IZP (Hoppins et al., 2016)) compared to B-form DNA (grey). Arrow shows the position of the lesion **(D)** Template-strand lesions are bypassed by TLS Pols, however as the lesion is exiting the active site, a steric clash may occur between a specific amino acid residue (i.e., Pinky Trigger) and the damaged template base to destabilize binding and further extension. Shown is the ternary *Sso*Dpo4 complex (PDB:ID 1JX4) with oxygen modelled in the 8-position of the guanine in the −3 position on the primer strand.

While most TLS studies have focused on the capability of TLS Pols to insert nucleotides opposite a template-strand DNA lesion, less focus has been placed on resumption of high-fidelity synthesis beyond the lesion. As TLS Pols are often inaccurate and less processive for synthesis opposite an undamaged DNA template, they are limited in their extension capabilities after lesion bypass (Vaisman and Woodgate, 2017). Limiting extension by TLS Pols requires a mechanism for substitution back to a HiFi Pol. The length of extension beyond the lesion by TLS Pols can vary, and likely has to do with the native processivity of the TLS Pol on DNA templates (Figure 5A), additional Pols in the vicinity of the primer/template junction (Figure 5B), the contacts that the TLS Pol active site residues make with the DNA substrate (Figure 5C), and possibly even structural impediments to extension (Figure 5D). After TLS has been completed, the TLS Pols extend to a position beyond the lesion, which is now outside of the Pol active site. At this point, structural characteristics of the TLS Pol can cause dissociation from the DNA substrate and initiate a substitution back to the HiFi Pol.

### Inherent Translesion Synthesis Pol Processivity

A primary reason for why TLS Pol synthesis is limited is due to low processivity on undamaged substrates (Figure 5A). TLS Pols have the structural features to allow bulky lesions into the active site (Figure 1C), but they dissociate readily from undamaged DNA (Raper et al., 2016). The large, tolerant active sites of TLS Pols are optimal for TLS but make the TLS Pol inherently less stable, less processive, and more mutagenic on undamaged substrates. “Extender” Pols have slightly increased processivity compared to “inserter” Pols but remain limited in synthesis capability once far enough downstream from the lesion. Recent structural studies have identified the position of the N-terminal domain-palm domain linker in the catalytic subunit of human Pol ζ as paramount in allowing the extender to tolerate distorted templates (Malik et al., 2020). Beyond active-site recognition of the DNA damage, Pol ζ processivity is greatly reduced. This finding is supported *in vivo* by examination of human Pol ζ mutational signatures in extension past damaged DNA. Recently, human Pol ζ was found to extend the nascent strand of DNA roughly 30 nucleotides from BPDE-induced damage (Table 1) (Suzuki et al., 2021). The mutational frequency data also indicated that Pol ζ may be recruited multiple times during extension, even after Pol δ has performed some synthesis. Therefore, further research needs to be performed to confirm the exact mechanism of limiting “extender” Pols in TLS. Adding to this complexity are the shared subunits (PolD2 and D3) between replicative Pol δ and extender Pol ζ. It also remains unclear the exact position of the *exchange* or *switch* back following Pol ζ extension, and whether or not this position is the same for extension past all types of DNA lesions.

**TABLE 1:** The position of substitution back to high fidelity synthesis.

DNA lesion	Species	Inserter	Extender	*Exchange* or *Switch* back position	Reference
Benzo(a)pyrene diol epoxide(BPDE)-dG	*Hs*	Pol κ	Pol ζ	+30	Suzuki et al. (2021)
Cyclobutane pyrimidine dimer (CPD)	*Hs*	Pol η	N/A	+2	Kusumoto et al. (2004), McCulloch et al. (2004)
8-oxoguanine (8-oxoG)	*Sso*	Dpo4	N/A	+3	Cranford et al. (2020)
N^2^-dG-peptide	*E. coli*	Pol IV	N/A	+3	Minko et al. (2008)

Direct contacts between the TLS Pol active site and the damaged DNA template also contribute to the position of the hand-off to a replicative Pol. Likely contributing to processivity, the ability of a TLS Pol to interact with the lesion as it leaves the active site can dictate how many additional nucleotides the Pol will synthesize before dissociating from DNA. For certain Pols, such as human Pol η and its bypass of UV-induced TT-dimers, the TLS mechanism has been thoroughly examined. Crystal structures of Pol η in complex with CPD-containing DNA have shown that the Pol η active site can easily accommodate two template-strand nucleotides (Biertumpfel et al., 2010). Having the dimer within the Pol η active site is crucial to maintaining interaction with the DNA and allows for efficient TLS across dimerized lesions in eukaryotes (Prakash and Prakash, 2002). During bypass of a CPD, it has been shown that Pol η inserts nucleotides opposite the dimer, extends two positions beyond the lesion, and then destabilizes from DNA (Kusumoto et al., 2004; McCulloch et al., 2004). When Pol η is three nucleotides from the template-strand dimer it becomes deprived of these stabilizing contacts (Biertumpfel et al., 2010). This correlates with the position at which the HiFi Pol is able to resume synthesis after TLS. For bypass of this particular DNA lesion in humans, an “extender” Pol is not necessary, confirming +2 nucleotides past the lesion as the position for the HiFi substitution (Table 1).

### Equilibrium Competition for DNA Binding

The concentrations of TLS Pols are generally kept lower than HiFi Pols to prevent their equilibrium association and low fidelity synthesis. In fact, altered expression of the TLS Pols have been linked with increased mutation rates (Pavlov et al., 2001; Qi et al., 2012; Sasatani et al., 2017), correlated with various cancers (O-Wang et al., 2001; Albertella et al., 2005b; Flanagan et al., 2007; Wang et al., 2010), and give rise to chemotherapeutic resistance (Saha et al., 2021). There is evidence that TLS Pols are cell cycle regulated under normal conditions, peaking in G2 phase to facilitate any required TLS prior to cell division (Waters and Walker, 2006; Plachta et al., 2015; Sobolewska et al., 2020). Even reorganization of Pol δ in eukaryotes from a three to four subunit enzyme (Zhang et al., 2007; Lee et al., 2019) or exchanging with Rev3/7 to form Pol ζ (Rizzo et al., 2018) can impact activity. The return of the p12 subunit to reestablish a four subunit Pol δ is influential in providing greater processivity and strand displacement activity possibly reserved for other DNA repair pathways (Meng et al., 2010; Lin et al., 2013).

Several eukaryotic TLS Pols, including Pol η (Tomicic et al., 2014), Rev1 (Uchiyama et al., 2015), and Pol κ (Velasco-Miguel et al., 2003) are upregulated after specific DNA damage, however the prototypical upregulation of TLS Pols occurs during the SOS response in bacteria to overcome substantial DNA damage and induce DNA mutagenesis for survival (Kenyon and Walker, 1980; Napolitano et al., 2000; Yeiser et al., 2002). Deubiquitination of PCNA may also serve to recruit HiFi Pols back after insertion/extension events, although this may be more influential in the yeast system (Zhuang et al., 2008) compared to mammalian systems (Niimi et al., 2008; Brown et al., 2009). Therefore, with all of these potential expression changes occurring normally during the cell cycle and more specifically in response to DNA damage and in cells with multiple TLS Pols, it is likely that even small changes in the cellular multiequilibrium will affect Pol binding and selection (Figure 5B).

### Altered DNA Conformation

Some TLS Pols have active sites evolved for specific lesions (Sale et al., 2012; Sale, 2013) either to recognize the lesion itself or the alternative conformation of the primer-template DNA duplex induced by the lesion. Of the most common lesions, 8-oxoG does not induce significant structural duplex distortion (Plum et al., 1995), unlike that for an abasic site (Bignon et al., 2016) (Figure 5C). Other lesions such as benzopyrene dG adducts (Menzies et al., 2015), CPDs (Mcateer et al., 1998), or cis-Pt (Hartog et al., 1983) also induce major structural distortions (Pages and Fuchs, 2002; Lukin and De los Santos, 2006; Zhao et al., 2012). After insertion of a base across these lesions, the “inserter” Pol will move to the +1 position. From there, the “inserter” or “extender” will continue to synthesize downstream of the lesion. The structural perturbations that these Pols encounter past the lesions has not been studied in great detail and provide a plausible model for binding destabilization that limits further extension. Interestingly, the extender Pol ζ includes structural features to tolerate lesion-distorted DNA upstream (Malik et al., 2020).

### Steric Impediments to Extension

While the aforementioned contacts with DNA stabilize the Pol during TLS, some interactions between the Pol active site and the lesion can promote dissociation from the substrate. Recent studies on the bypass of 8-oxoG lesions by *Sso*Dpo4 have indicated that this Pol is able to insert a nucleotide opposite the lesion and extend three bases beyond the lesion before becoming catalytically inefficient (Table 1) (Cranford et al., 2020). This coincided with the exact position (+3) that the replicative Pol B1 is capable of reengaging and efficiently extending the primer to resume high fidelity synthesis. Upon structural examination of Dpo4 in complex with DNA, specific residues of the little finger domain may be directly clashing with the 8-oxoG lesion as it exits the active site. This hypothetical “pinky trigger” may dictate the position where HiFi synthesis resumes for the archaeal system (Figure 5D **)**. The ability of TLS Pols to sense the template lesion outside of the active site may destabilize binding of the TLS Pol downstream and provide an opportunity for a HiFi Pol to resume synthesis.

## Therapeutic Strategies Targeting TLS Steps

Polymerase inhibitors have become part of the ever-growing list of chemotherapeutics. As accurate and complete replication of DNA is vital to cellular growth and viability, inhibition of this process can block growth and promote apoptosis of aberrant cells. By specifically targeting cancer cells with molecules designed to impede DNA replication, one could limit tumor growth. Chain-terminating nucleoside analogs are among the most popular in this class of therapeutics (Berdis, 2017). These nucleotide mimics are inserted by Pols to a nascent strand of DNA during replication and promptly conclude extension of that strand. The potential impact of these chain terminators can be escalated when combined with DNA-damaging agents. TLS Pols are known to bypass lesions generated by platinum chemotherapeutics (Albertella et al., 2005a) and are upregulated in multiple cancer cell lines (Tomicic et al., 2014). The overzealous activity of these TLS Pols desensitizes cancer cells to chemotherapeutics by enabling cell survival amid DNA-damaging conditions. By inhibiting these TLS Pols and preventing insertions opposite chemotherapy-induced lesions or other steps in the TLS process, the efficacy of the treatment is amplified overall.

In accordance with this principle, alternate methods of TLS Pol inhibition would suffice for ameliorated chemotherapy. Pol-Pol and PCNA-Pol contacts that are involved in TLS Pol recruitment, stabilization, and substitutions make ideal targets for cancer drug research (Altieri and Kelman, 2018). TLS in humans across cisplatin-induced intrastrand crosslinks is a three-substitution mechanism requiring Pol η as the inserter, Pol ζ as the extender, and Rev1 as a scaffold. Recently, JH-RE-06, a small molecule inhibitor of this bypass, has been shown to dimerize Rev1 and prevent Pol ζ from binding (Wojtaszek et al., 2019). Without an efficient extender, TLS opposite Pt-GG lesions is reduced, and cisplatin becomes more effective at counteracting tumors. JH-RE-06, along with other small molecule inhibitors of protein-protein interactions, serve to block a specific element of TLS mechanisms in order to minimize obstructions to DNA-damaging agents (Wojtaszek et al., 2019; Dash and Hadden, 2021).

## Discussion

All of the examples described in the Postlesion: Resuming High Fidelity Synthesis, a Final Substitution section are mechanisms used to limit synthesis by a TLS Pol after insertion to maintain downstream genome fidelity. The same structural features of TLS Pols that allow bulky lesions to enter the Pol active site are the features that make TLS Pols inaccurate and unstable on undamaged DNA substrates. TLS Pols are more mutagenic on undamaged templates than on suitable damaged substrates; so strict regulation outside of a specific TLS insertion is necessary. Thus, TLS Pols must insert nucleotides opposite the lesion, extend beyond the lesion, and then hand the DNA back to a HiFi Pol at a position that is both favorable to TLS Pol dissociation and subsequent HiFi Pol extension. Integral to the positioning of the resumption of HiFi synthesis are direct Pol-DNA, Pol-PCNA, and Pol-Pol interactions. Pol-Pol binding has been shown to stabilize the replicating Pol throughout TLS (Cranford et al., 2017). Pol-PCNA contacts through PIP motifs, UBZ/UBM contacts, and Rev1 bridges are also known to improve Pol stability and processivity (Boehm et al., 2016b; Acharya et al., 2020). These precise interactions that provide stability during TLS may also be involved in regulation of steps after TLS insertion. Further experimentation needs to be performed to identify the exact substitution mechanism(s) and steps that occur during lesion bypass.

Many *in vitro* experiments examine the TLS lesion bypass capability of truncated core TLS Pols in isolation. However, these absent regions are precisely the ones that facilitate Pol-Pol and PCNA-Pol binding and are needed to examine the entire TLS process. Full-length TLS and HiFi Pols, in the presence of PCNA and a suitable “extender” Pol will allow for interactions that may influence extension beyond the lesion, thus providing confidence in the kinetic characterizations required to determine a position where high fidelity synthesis resumes. Ultimately, translesion synthesis needs to be rapid, but distributive, to limit successive nucleotide incorporations so that replicative Pols can resume high fidelity synthesis and recouple with the replisome. The entire TLS mechanism, from the initial HiFi Pol stalling, “inserter” Pol recruitment, and TLS Pol(s) selection to the second (and possibly third) Pol substitutions to an “extender” Pol or HiFi Pol, respectively, remains to be fully understood. Location of the substitution positions and identification of the kinetically favored TLS mechanisms for bypass of DNA damage has direct applications in drug discovery. Future experiments should seek to expand our understanding of the complete TLS process to identify additional therapeutic targets within TLS.
